# Synovial sarcoma of female urethra: a case report and review of the literature

**DOI:** 10.1186/s13000-023-01367-z

**Published:** 2023-07-03

**Authors:** Wei Cui, Yuan-Jian Liao, Peng Su, Hua Yang, Neng Zhang

**Affiliations:** 1 Department of Urology, The Affiliated Hospital of Zunyi Medical University, No.149 Dalian Road, Zunyi, Guizhou China; 2 Department of Pathology, The Second Affiliated Hospital of Zunyi Medical University, Zunyi, Guizhou China

**Keywords:** Case report, Diagnosis, Urethra, Synovial sarcoma

## Abstract

Synovial sarcoma (SS) is a rare malignant soft tissue sarcoma that originates from primitive mesenchymal cells with epithelial differentiation potential. It is most commonly found in the limbs and trunk. In the urinary system, it is mostly found in the kidneys. However, synovial sarcomas originating from the external urethra are extremely rare. Only one case of synovial sarcoma arising from the vulvar urethral orifice has been reported previously, and we report a second case of synovial sarcoma of the urethral orifice. In addition, a total of 16 vulvar synovial sarcomas were identified and the literature are analyzed in this report reviews from 1966 to the present.

**Supplementary Information:**

The online version contains supplementary material available at 10.1186/s13000-023-01367-z.

## Introduction

Synovial sarcoma (SS) is a rare malignant soft tissue sarcoma, representing soft tissue sarcoma (STS) with an indeterminate direction of differentiation. SS is very rare, with an incidence of approximately 1.42 cases per million adults [1]. As such, here are few reported cases of SS, which originate from primitive mesenchymal cells with epithelial differentiation potential and whose pathophysiology has not been clearly elucidated. Depending on the primitive location of SS, the most common site is the lower limbs, especially the knee region, but also the whole body, including the upper limbs, head and neck, abdominal wall or retroperitoneum [2, 3]. However, primary urethral synovial sarcoma is extremely rare in previous reports. Here, we report a case of primary urethral synovial sarcoma and evaluate the available literature.

## Case report presentation

**Fig. 1:**
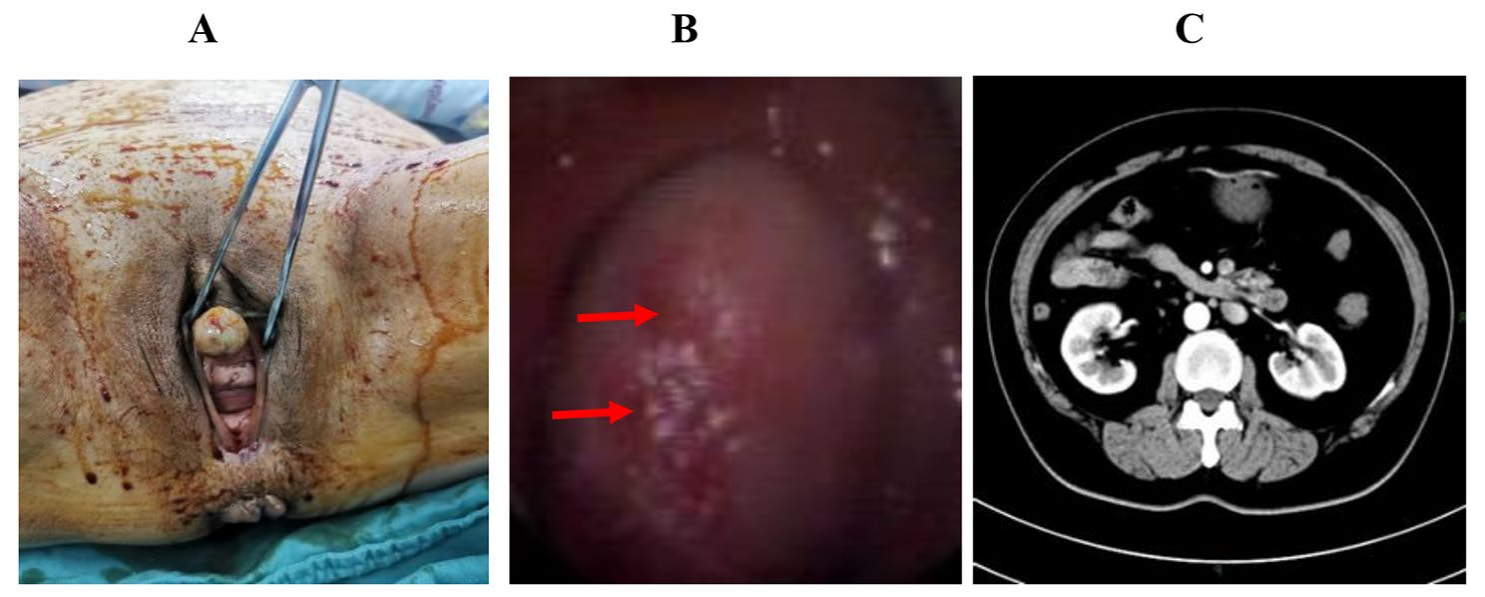
Pre-operative examination. **A** The gross appearance of an external urethral synovial sarcoma; measuring approximately 3.0 cm × 4.0 cm, with completed coat on the external genitalia. **B** Cystoscopy revealed: a large mass measuring approximately 2.5cmx2.0 cm with ulceration and oozing on the right side of the external urethral opening. **C** Plain CT scan shows no bilateral ureteral dilatation and no dense shadows in the bilateral ureteral travel area

**Fig. 2:**
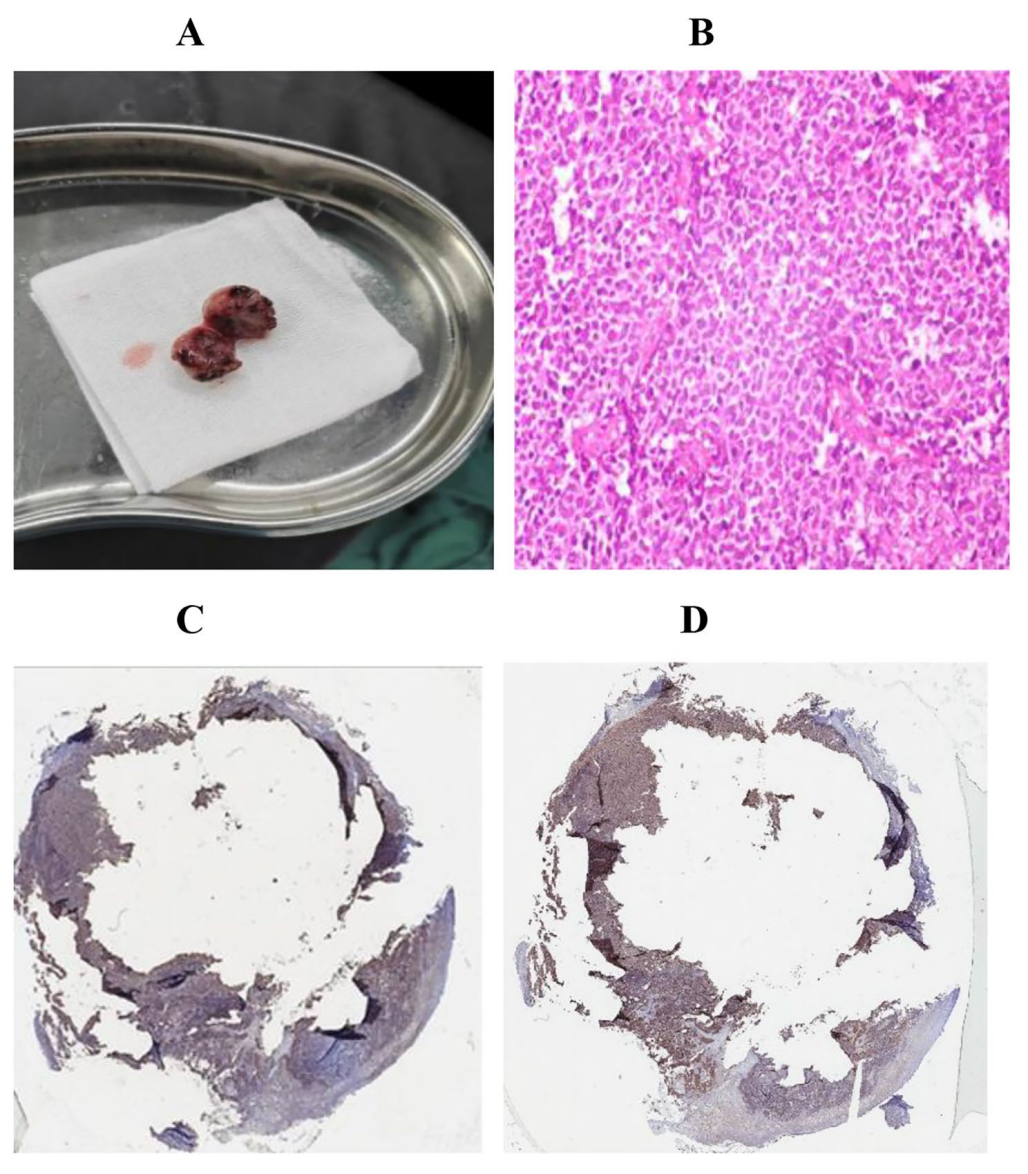
Postoperative pathological and immunohistochemical staining results. **A** Mass specimen. **B** Pathological images. **C** Tumor cells were positive for Recombinant Cluster of Differentiation 99 (CD99). IHC 1×. **D** Tumor cells were positive for B-cell leukemia/lymphoma 2 (Bcl-2) protein. IHC 1×

A 60-year-old female patient was admitted to the hospital with main complain “urethral bleeding detected after activity 15 days ago”. A painless mass, its size about 3.0 cm × 4.0 cm, with completed coat on the external genitalia (Fig. 1A)and the rest of the physical examination was normal when physical examination. Normal urine erythrocytes (++), routine blood, electrolytes, liver and renal function, coagulation, thyroid function, carbohydrate antigen 19 − 9 (CA19-9) and alpha-fetoprotein (AFP) among laboratory tests. Any significant abnormalities were not found in electrocardiogram and chest CT. A mass was approximately 2.5 cm × 2.0 cm with ulceration and oozing on the right side of the external urethral orifice (Fig. 1B) by cystoscopy. Urological pelvic scan + enhancement + 3D CT showed no dilatation of the ureters bilaterally and no high-density shadow in the ureteral travel area bilaterally (Fig. 1C). Greyish grey-red tissue was found in the gross specimen of the postoperative tumour section (Fig. 2A). The pathological diagnosis was considered to be a malignant tumour of the external urethral orifice(Fig. 2B). Immunoprecipitation analysis results were as follows: CD99 (+) (Fig. 2C), Syn (local +), INI-1 (+), CD34 (-), CD56 (scattered +), CgA (-), CK (focal +), CK7 (-), CK-H (-), CK-L (focal +), Desmin (-), GATA3 (-), Ki-67 (60% +), LCA (-) MyoD1 (-), Vimentin (partial +), HBM45 (-), Melan-A (-), S-100 (-). The Immunoprecipitation analysis combined with pathological diagnosis was considered to be synovial sarcoma of the external urethral orifice. To further confirm the diagnosis, We sent the pathology section to the Department of Pathology of the Cancer Hospital of Sun Yat-sen University for consultation. The results of immunoprecipitation analysis at the Affiliated Cancer Hospital of Sun Yat-sen University were as follows: bcl-2 (++++) (Fig. 2D), CK8/18 (small amount +), p40 (small foci +), CK5/6 (-), p63 (small foci -). The result was positive for the SS18 gene break by FISH and, in combination with the Immunoprecipitation analysis results, the final diagnosis of synovial sarcoma of the external urethral orifice was confirmed. The patient had a good postoperative course and was discharged on postoperative day 6 without any complications.

## Discussion

Synovial sarcomas (SS) are malignant soft tissue sarcomas that originate from primitive mesenchymal cells with epithelial differentiation potential and represent soft tissue sarcomas (STS) with an indeterminate direction of differentiation. Synovial sarcomas account for approximately 13% of STS [4]. SS grows relatively slowly, usually occurs in patients aged 15–40 years, and affects both sexes equally [5]. The most common sites include the lower limbs (60%), particularly the knee region; followed by the upper limbs (23%), the head and neck region (9%), particularly in the retropharyngeal and parapharyngeal regions; and in the trunk (8.1%), the abdominal wall or retroperitoneum is most often involved [2, 3]. In the urinary system, synovial sarcoma occurs mostly in the kidney, and synovial sarcoma originating in the external urethral orifice is extremely rare. In the case we report, it occurred in the urethra of a 60-year-old woman. Previous reports have shown that SS tends to present as a painless mass and that the most frequent site of involvement is the knee of the lower limb [2], which is consistent with our systematic review in terms of symptoms, but in our case, it occurred in the external urethral orifice of an elderly female patient. To date, to the authors’ knowledge, a total of 16 cases of synovial sarcoma occurring in the vulva have been reported in 14 studies, with the youngest patient being 21 years old [6] and the oldest being 62 years old [7], and the mean age of the patients being 35.3 years (35.3 ± 12.0). Tumour size ranged from 0.8 [7] to 9 cm [6] (3.90 ± 2.51). 12 (75%) patients presented asymptomatic [6–12], which is consistent with previous views. 11 patients (68.7% with biphasic SS [6–8, 12, 13] of which 2 were hypofractionated biphasic SS [12] and the rest were monophasic). 12 (75%) patients underwent surgical resection [6–13] and 4 (25%) of them developed distant metastases [7, 8, 11, 12]. 10 of the 16 patients were followed for between 6 months [12] and 7.4 years [7], and only three died of tumour recurrence during the follow-up period [6, 7], as detailed in Supplementary Tables 1, Additional file.

Clinically, synovial sarcoma often presents in its early stages as a deep, painless mass with poorly defined borders and poor mobility, which may be free of any harmful symptoms, but as the tumor grows in size and compresses surrounding nerves and tissues in later stages, a range of signs and symptoms may develop. The most common symptoms are localized swelling, lumps, pain, restricted movement of adjacent joints and occasionally generalized malaise, fever and cachexia, due to systemic symptoms caused by the spread of the tumor. Unlike most sarcomas which present as a rapidly growing painless mass, synovial sarcomas grow slowly and may be preceded by pain or joint contracture [14]. However, patients with pain prior to swelling are not diagnosed earlier than patients without pain. According to Lawrence et al. [15], approximately half of patients with soft tissue sarcoma were diagnosed approximately four months after the onset of symptoms and another 20% were diagnosed six months after the onset of symptoms. This may be due to the slow growth and insidious nature of SS, the young age of the patient at presentation and the fact that the early stage of the tumor resembling a benign tumor is not taken seriously by the patient. A high proportion of synovial sarcoma cases are reported to be symptomatic for an average of 2–4 years, although in some rare cases this duration has been reported to be over 20 years [16]. This often results in patients having metastases as soon as they are diagnosed, with 50-70% of patients developing metastases [2]. The most common site of distant metastases is the lung [17], but also to lymph nodes, liver and bone [18], with massive pleural metastases being the main cause of death in synovial sarcoma. Survival rates for patients with synovial sarcoma without systemic therapy range from 40% to 60 [19]. A retrospective analysis by Krieg et al. showed [17] that important poor prognostic factors for SS included whether the tumor was > 5 cm, whether it was combined with metastases at diagnosis, histological grading, other co-morbidities and in- or marginal surgery of the lesion. Prognostically, synovial sarcomas aged < 20 years, with tumor diameters < 5 cm and better differentiation have a better prognosis. In contrast, those with metastases, tumors > 5 cm in diameter and poorly differentiated SS have a poorer prognosis.

Radiological investigations such as radiographs, ultrasound, computed tomography (CT) and magnetic resonance imaging (MRI) can be used as an initial assessment of SS. Ultrasonography can be used to understand the size of the mass, the site of invasion and to get a preliminary idea of the nature of the mass. CT and MRI examinations, in addition to showing the location, size and nature of the lesion, can be used by doctors to see if lymph nodes or lung metastases have occurred. However, a definitive diagnosis of SS requires a combination of pathology, molecular genetics and immunohistochemistry. Immunohistochemistry is an important tool in the diagnosis of synovial sarcoma. SS usually expresses low molecular weight keratins such as AE1/AE3, CK7/CK18/CK19 and possibly higher molecular proteins such as CK14 and CK17. Focal CK or EMA expression is usually expressed in 90% of synovial sarcomas [20]. S-100 is positive in approximately 23% of PDSS [21], but both neurogenic tumors and synovial sarcomas can show an S100 response, so they are easily misdiagnosed as neurogenic tumours. NY-ESO-1 shows high levels and diffuse expression in most synovial sarcomas, with a high specificity [22], which is also a target for T-CRT action. At the molecular level, the t(X;18) (p11.2; q11.2) chromosomal translocation, and the resulting SYT-SSX fusion gene, are present in almost all SS. The fusion of SYT-SSX1 and SYT-SSX2 disrupts gene function, leading to activation of the proto-oncogene or repression of the oncogene, allowing the cells to become cancerous [23]. PCR and other techniques can be used to detect fusion genes, but RT-PCR is more sensitive, and this is an important tool for confirming SS diagnosis.

The predominant treatment for SS is surgical resection. For tumors without lymph node metastases, tumors less than 5 cm in diameter and those located superficially, wide excision is the mainstay to obtain a suitable negative margin [24]. Compared to other soft tissue sarcomas, synovial sarcoma is more sensitive to chemotherapy, and chemotherapy, especially high-dose chemotherapy, has been widely used in the treatment of synovial sarcoma in recent years. Currently, adriamycin is still used as the first-line treatment in chemotherapy for STS [25]. However, the commonly used chemotherapy regimen for SS is based on doxorubicin combined with isocyclophosphamide. According to a randomised controlled trial by Judson et al., doxorubicin combined with isocyclophosphamide had a higher response rate (26.5% vs. 13.6%) and median PFS (7.4 months vs. 4.6 months) compared to adriamycin alone [26]. The improved response rate associated with combination chemotherapy may help improve patient survival time. Neoadjuvant or adjuvant radiotherapy is recommended for SS with tumours > 5 cm in diameter or where neurovascular structures or bone need to be preserved [27]. Radiotherapy has been shown in the past to be an important tool for improving local control and preventing recurrence. A study of synovial sarcoma of the head and neck showed that patients treated with surgery combined with adjuvant radiotherapy had higher survival rates and lower recurrence rates compared to patients treated with surgery, chemotherapy and radiotherapy alone [28]. However, preoperative chemotherapy can increase the risk of wound complications, while postoperative radiotherapy can lead to fibrosis and joint stiffness, resulting in poorer long-term functional outcomes [29].

In order to improve the efficacy of anti-tumour therapy and to reduce tissue damage, scholars have proposed targeted therapy and immunotherapy. The greatest advantage of targeted therapy is that it is highly selective, causes less damage to normal tissues and results in far fewer adverse effects than conventional radiotherapy and chemotherapy. Pazopanib is an oral multi-targeted tyrosine kinase inhibitor that directly targets receptor tyrosine kinases (RTKs), vascular endothelial growth factor (VEGFR) 1/2/3, platelet-derived growth factor (PDGFR) α/β and KIT, thereby blocking tumour growth and inhibiting angiogenesis [30]. A randomised controlled phase III study from Japan showed that the median PFS was significantly longer in the pazopanib treatment group, 24.7 weeks vs. 7.0 weeks, but did not significantly improve median OS, 15.4 months vs. 14.9 months, when receiving oral pazopanib 800 mg compared with placebo [31]. One study found that hyperactivation of IGF1 and insulin receptors (IGF1R / InsR) maintained AKT activation and pazopanib resistance in a CME-1 cell line, and that pazopanib resistance could be overcome by combination therapy with the dual IGF1R / InsR inhibitor BMS754807 [32]. Regorafenib is a small molecule oral anti-angiogenic drug that inhibits VEGFR, TIE-2, PDGFRs, RAF, KIT and FGFR [33]. Clinical studies have shown that regorafenib for synovial sarcoma significantly prolonged PFS compared to placebo, 5.6 months vs. 1.0 month [34]. In the field of immunotherapy, by far the most promising therapeutic approach is engineered T-cell (expressed in 80% of SS patients) therapy against NY-ESO-1 cancer/testis antigen [35]. According to Robbins et al. long-term follow-up, 11 of 18 patients (61%) with advanced synovial sarcoma showed tumour regression after treatment with TCR-T, with 3- and 5-year survival rates of 38% and 14%, respectively [36]. A phase I trial at the University of Texas MD Anderson Cancer Center in Houston showed that the novel TCR-T cell therapy ADP-A2M4 therapy showed the greatest promise in synovial sarcoma, with a disease control rate of approximately 90% [37]. There are still many more clinical studies in SS for NY-ESO-1 in the trial phase, but based on the available findings, exciting early results have been shown.

## Conclusion

Synovial sarcomas are very rare, even rarer when they originate in the external urethra. Because of its location, synovial sarcoma of the external urethra can easily go unnoticed by being misdiagnosed as other vulvar tumours. The diagnosis of synovial sarcoma of the external urethra requires pathology and immunohistochemistry, with further tests such as special stains and molecular pathology for the auxiliary and differential diagnosis if necessary.

## Electronic supplementary material

Below is the link to the electronic supplementary material.

Supplementary Material 1

## Data Availability

The original contributions presented in this study are included in the article/supplementary material, further questions can be directed to the corresponding author.
