# Supplementary material for: Synovial sarcoma of female urethra: a case report and review of the literature
Source: Diagn Pathol. 2023 Jul 3;18:78. doi: 10.1186/s13000-023-01367-z (PMC10316565; doi:10.1186/s13000-023-01367-z)
Supplement: Supplementary file 1 — Supplementary Material 1 [file 13000_2023_1367_MOESM1_ESM.docx]

**TABLE 1. Review of characteristics of Synovial sarcoma**

| **Case No** | **First author/Year** | **Age(years)**  **/Sex** | **Clinical Symptoms** | **Site** | **Size**  **(cm)** | **Diagnosis** | **follow-up (months)** | **Immunohistochemistry** | **Molecular** | **Condition** |
| --- | --- | --- | --- | --- | --- | --- | --- | --- | --- | --- |
| 1 | Nielsen et al./1996(4) | 30/F | Asymptomatic | Vulva | 2 x 1.9 x 1.6 | Biphasic SS | 12 | Epithelial cells-CK+ spindle cells-vimentin+ | NA | Alive |
| 2 | Nielsen et al./1996(4) | 37/F | Asymptomatic | Vulva | 1.2 | Biphasic SS | 48 | Epithelial cells-CK+ spindle cells-vimentin+ | NA | Alive |
| 3 | Ambani et al./2006 (5) | 33/F | Asymptomatic | Vulva | 6.5 x 4.2 x 3.5 | Monophasic SS | 8 | Vimentin+ ，CK focally+ | RT-PCR:  SS18/SSX | Alive |
| 4 | Holloway et al./2007 (6) | 50/F | Asymptomatic | Vulva | 4.2 x 2.5 x 1.4 | Monophasic SS | 14 | imentin, Bcl 2 diffusely+;  CK, EMA focally+ | NA | Alive |
| 5 | White et al./ 2008(7) | 33/F | Asymptomatic | Vulva | 6.5 x 4.2 x 3.5 | Monophasic SS | NA | CK diffusely+, vimentin focally+ | SS18-SSX2 | Alive |
| 6 | Sumathi et al./ 2011(8) | 24/F | Asymptomatic | Vulva | 7.5 | PD biphasic SS | NA | CK, EMA diffusely+ in epithelial component,  focally+ in spindle cell  component;  Bcl-2, CD99, focally+ | RT-PCR and SEQ:  SS18/SSX1 | NA |
| 7 | Sumathi et al./ 2011(8) | 29/F | Clinical impression  of lipoma | Vulva | 2.2 | PD biphasic SS | 6 | Diffuse CK+,EMA+, in epithelial component,  focal+ in spindle cell component; Bcl-2+ and CD99- | RT-PCR and SEQ:  SS18/SSX1 | Alive |
| 8 | Sumathi et al./ 2011(8) | 27/F | Clinical impression  of vulval cyst | Vulva | 1.5 | Monophasic SS | NA | Focal CK+; EMA+,  Bcl-2+, CD99- | RT-PCR and SEQ:  SS18/SSX2 | NA |
| 9 | Asher et al./ 2011(9) | 28/F | Swelling of the  left vulva | vulva | 2.2×1.5×1.0 | Biphasic SS | 36 | AE1/3+,CD 117+, vimentin+, LCACD68-,NSE-、CD34-、 CAM5.2-、SMA-、desmin-,S100- | SYT-SSX | Alive |
| 10 | Kawauchi et al./ 2012(10) | 21/F | Asymptomatiic | vulva | 9x8x7 | Biphasic SS | 48 | vimentin+, CD99+, Bcl-2+,NSE+ ,CD34- | NA | Died |
| 11 | Kolin et al./ 2020（11） | 50/F | Asymptomatic | vulva | 4.2 | Monophasic SS | 88.8 | SS18-SSX+, SSX+, GFAP+，pan-keratin+ ，PAX8-, ER-, CAM5-. AE1/AE3-, EMA-, S100-, SMA-, desmin-, synaptophysin-, HMB-45-, melan-A-, chromogranin- | FISH:  SS18 rearrangement | Alive |
| 12 | Kolin et al./ 2020（11） | 62/F | Clinical impression  of Bartholin cysts | vulva | 6 | Biphasic SS | 55 | SS18-SSX+, SSX+,  EMA+, TLE1+,  p53 (wild-type)+；PAX8-,  ER-, PR-,AE1/AE3-, WT-1 - | QncoPanel:  SS18-SSX1 fusion | Died |
| 13 | Kolin et al./ 2020（11） | 47/F | Asymptomatic | vulva | 0.8 | Biphasic SS | NA | SS18-SSX+,SSX+, TLE1+,ER(30%,weak)-,p63- | FISH:  SS18 rearrangement | NA |
| 14 | Kolin et al./ 2020（11） | 46/F | Asymptomatic | vulva | 2.5 | Biphasic SS | NA | SS18-SSX+,SSX+, TLE1+,CAM5.2+， CD99+， PAX8+，ER-, PR-,GATA3-, CD34-,p63-,GFAP-,  desmin-, SOX1- | FISH:  SS18 rearrangement | NA |
| 15 | Kolin et al./ 2020（11） | 26/F | Asymptomatic | vulva | 2.1 | Biphasic SS | 36 | SS18-SSX+,SSX+, EMA+，PAX8-, ER- | FISH:  SS18 rearrangement | Died |
| 16 | Kolin et al./ 2020（11） | 23/F | Asymptomatic | vulva | NA | Biphasic SS | NA | NA | RT-PCR:  SS18-SSX fusion | NA |

F, Female; PD, poorly differentiated; SS, synovial sarcoma; SEQ, sequencing; RT-PCR, reverse transcription polymerase chain reaction, NA, Not available.
